# Beware the Little Foxes that Spoil the Vines: Small Inconsistencies in Clinical Data Can Distort Machine Learning Findings

**DOI:** 10.26502/fjhs.348

**Published:** 2025-09-11

**Authors:** Abdolvahab Khademi, Mark S. Tuttle, Qing Zeng-Treitler, Stuart J. Nelson

**Affiliations:** 1 Biomedical Informatics Center, George Washington University, Washington, DC; 2 Orinda, CA

**Keywords:** Information theory, International Classification of Diseases, Coding, Noise, Data Quality

## Abstract

It is well known that Electronic Health Records (EHR) data contain inconsistent and inaccurate data, the effect of which on predictive model performance and risk/benefit factor identification are often neglected. This study investigates how varying levels of random and non-random binary differences, often referred to as “noise”, affect modeling tools, such as logistic regression, support vector machines, and gradient boosting models. Using curated data from the All of Us database, we simulated different noise levels to mimic real-world variability. Across all models and noise types, increased noise consistently reduced classification accuracy. More importantly, noise diminished the variance of variable impact scores while leaving their means unchanged, suggesting a muted ability to identify key predictors. These findings imply that even modest noise levels can obscure meaningful signals. Measures like accuracy and hazard ratios may thus be misleading in noisy data contexts. The consistency of effects across models and noise mechanisms suggests this issue stems from inherent data variability rather than model brittleness, with broad implications for EHR data analyses.

## Introduction

In real-world data, such as that found in electronic health records (EHRs), a level of apparent inconsistency or “noise” in the data appears to be inevitable [1–5]. Patients with highly similar clinical conditions often have different free text notes and structured data in different healthcare systems and from different time periods [6–10]. This situation has a variety of causes including variance in clinical care, differing interpretations of the history, variance in data entry or extraction process, ambiguity in standard terminologies, and evolution in the terminologies. Additionally, biological variability is a feature of the evolutionary process, and our categories of clinical conditions (diagnoses) uncertain. It is unrealistic to think that the record of a person’s condition will be described and recorded reproducibly. Coding variation, on the other hand, is a well-known problem [11–15]. While human coders can make mistakes, they are not completely responsible for the observed differences. Sometimes physicians’ notes are incomplete or are ambiguous when describing the clinical diagnoses [16]. Additionally, coding rules may lack clarity [17].

All these variations, which we will refer to as noise, stemming from measurement errors, missing information, subjective assessments, and population variability, are often difficult to detect. Completely clean datasets are rare. As a result, the level of noise is frequently unknown and overlooked. This situation introduces fundamental limits to the accuracy and reliability of models used to determine disease causes or make clinical predictions, even when advanced analytical methods are applied. To address the limitations of coded data, many research projects use natural language processing (NLP) methods to identify diagnoses, signs, and symptoms from accompanying text [18–24]. The ability of the NLP tools to identify significant text varies but rarely reaches 95% in both sensitivity and specificity. Like human coders, NLP tools also face the challenges of documentation quality and ambiguity in coding rules. Furthermore, NLP tools are typically trained and calibrated using human-annotated data, which also contains human errors [25, 26].

When working with large amounts of EHR data in clinical research, we often perform extensive data preprocessing to assemble a relatively “clean” dataset for a given project. In such endeavors, the focus is typically placed on the main observables and an outcome, while relying on existing coded data for the identification of any important covariates. One underlying belief or hope is that a small amount of irregularity will not affect the overall study outcome and that the law of large numbers will outweigh many concerns with the data.

Without a gold standard “clean” dataset, it is impossible to confirm this belief or assess the likelihood that this hope will prevail. Indeed, we have little evidence of the effect of noise in the predictors on the accuracy and utility of the findings. In observational research, we do not know how much variability will affect our results.

As part of a larger project to improve EHR data quality, we investigated the effect of noise on predictive modeling performance and findings derived from the predictive models. To measure the effect, we carried out a simulation by adding varying amount of noise to a real-world dataset postulated to be accurate. To increase the generalizability of our machine learning results, we employed three algorithms (logistic regression, support vector machine classifier, and gradient boosting). To the best of our knowledge, such experiments have not been previously carried out, despite the increasing concern regarding replicability of biomedical research [27–29].

## Background

The Shannon Channel Capacity Theorem, introduced by Claude Shannon in 1948 [30] defines the theoretical limit for transmitting information over a noisy channel using error-correcting methods. The theorem states that if the rate of information transmission R is less than the channel capacity C, then it is possible to design coding schemes that reduce the probability of error to an arbitrarily low level. In essence, reliable communication is achievable so long as the transmission rate stays below the channel’s capacity. On the other hand, if R>C, no coding method can ensure reliable transmission. There will always be a non-negligible error rate, which grows as the transmission rate increases. Therefore, when the rate exceeds the channel’s capacity, accurate information transfer cannot be guaranteed. The case where R=C is not fully addressed by the theorem. This principle has important implications in the context of medical data. Unlike engineered communication systems, medical data is highly complex and inherently noisy.

A review of the literature reveals several studies that examine the impact of random noise on the performance of machine learning algorithms [31]. In a simulation study, Xiao and Higgins introduced varying levels of random noise into the data and found that as noise increased, the strength of the relationship between covariates and outcomes (measured by regression weights) weakened [32]. They cautioned that adding more covariates can amplify the noise, potentially overwhelm the signal and lead to invalid or inconsistent results. Plevris et al. simulated different levels of Monte Carlo Markov Chain Gaussian noise and observed that most predictive modeling metrics deteriorated as noise increased.^33^ Their findings emphasize the importance of accounting for covariate inaccuracies when modeling the relationship between predictors and outcomes. Kolmar et al. studied the effects of experimental error, modeled as Gaussian noise, on RMSE and R-squared metrics across several machine learning models.^34^ They found that both metrics worsened as the level of random variation increased. None of these studies were conducted within the biomedical domain, nor did they compare different types of noise mechanisms. Moreover, they primarily focused on prediction accuracy rather than feature contributions (such as risk factors), which are often of greater importance in biomedical research.

## Methods

We extracted a real-world dataset from the US NIH All of Us project [35]. In the prediction models, the outcome variable was the recording of an Alzheimer’s Disease or Related Dementia (ADRD) condition, and the independent variables were comorbidities represented by International Classification Diseases (ICD) codes. Since ICD-codes assignments are known to be very inconsistent [36], the ICD-9CM and ICD-10CM codes were aggregated into broader categories referred to as code blocks [37].

### ADRD Condition Definition

We used a broad definition of ADRD to include different related mental conditions, including dementia, cerebral degeneration, and senile dementia and subtypes [38]. These conditions were identified using the ICD 9 and ICD 10 codes. Table A-1 in Appendix A lists the ICD 9 and ICD 10 codes and their description to identify ADRD conditions.

### Code Blocks

Code blocks are binary variables that represent conditions with a common component as designated by their ICD 10 codes. (01= presence of condition; 0 = absence of condition) For example, conditions associated with ICD 10 codes A00 through A09 are collectively described as intestinal infectious diseases and grouped into Code Block 1, and conditions in the ICD 10 code range A15 through A19 are grouped into Code Block 2 associated with tuberculosis. Table A-2 in Appendix A presents ICD 10 code ranges grouped into Code Blocks with terms describing the category.

### Data

From the All of Us database (with Controlled Tier access), a cohort of patients with age 55 years and older were selected. Data before January 1, 2000, were removed due to sparsity and inconsistency. In the cohort, cases are patients with an ADRD diagnosis. The control group was randomly sampled from those without ADRD diagnosis at 1:1 ratio (total n = 6,794). Data on predictors were collected before the first ADRD diagnosis for the cases and before the last visit for the controls. We converted ICD9 to ICD10 codes using the General Equivalence Mapping Tables provided by the Centers for Disease Control, and then mapped the ICD10 codes to their appropriate code blocks.

### Simulated Noise

In addition to the actual data retrieved from All of Us, we simulated 5%, 10%, 15%, 20%, 25%, and 30% of noisy data of the code blocks (i.e., the features) to study the effect of noise on the prediction of ADRD. In simulation studies investigating the effect of noise on machine learning algorithm performance, noise can be added as perturbation either to the features (attribute noise) or the outcome (class noise), or both. In our study, we chose to inject noise into the features and keep the outcome unmodified.

Our noise generation process was modelled on three different types of missing values mechanisms from missing value research literature as described by Rubin [39]. Noise completely at random (NCAR) refers to noises that are not related to collected data (observed variables) or outside the collected data (unobserved variables). NAR (Noise At Random) refers to the noises related to observed data but not noises themselves. NNAR (Noise Not At Random) refer to noises related to a hidden variable (which could be the noise itself).

In NCAR generation, we generated random noises by randomly switching a variable’s value from 0 to 1 or 1 to 0. In the NAR scheme, we simulated noise in a subset of the data that was constrained by an observed variable. Specifically, we randomly separated features into “changeable” and “unchangeable’ subsets. We then randomly flipped the values of the changeable features for the instances with the highest sum of the “unchangeable’ values. In the NNAR scheme, we generated noise by randomly changing the 0s into 1s. Because in the changed data the original 0s are hidden, this is considered to be not at random. We varied the amounts of noises as different proportions of the data: 5%, 10%, 15%, 20%, 25%, and 30%. Overall, we generated 18 simulated datasets using 3 random noise generation mechanisms with 6 proportions.

## Analysis

We next applied three machine learning methods to the data for prediction of the binary outcome (ADRD). The effect of random noise on model performance was measured by noting the potential changes in area under the receiver operating characteristic curve (AUC) and accuracy and feature weights or impact scores across the 18 simulation experiments.

In each experiment, we used logistic regression, gradient boosting (XGBoost), and linear support vector machine (SVM) models in Python to predict ADRD as a function of code blocks. We then compared the model performances to evaluate how the noise mechanisms affected the AUC and impact scores.The impact score is a measure of the contribution of a variable to the model. It quantifies the impact of an individual feature on the target outcome and is intended to quantify the explanatory power of features in linear and nonlinear machine learning models [40]. The impact scores in logistic regression with binary predictors are equal to the coefficients. In that sense, one could think of impact scores as a generalization of the notion of the coefficients seen in logistic regression. After computing the scores for each variable in each model, we computed the means for each method across noise proportions and performed a repeated-measures analysis of variance (RM-ANOVA) to investigate if noise proportions have significant effects on variable impact scores in the three machine learning algorithms.

## Results

### Model Performance Metrics

**Figure 1:**
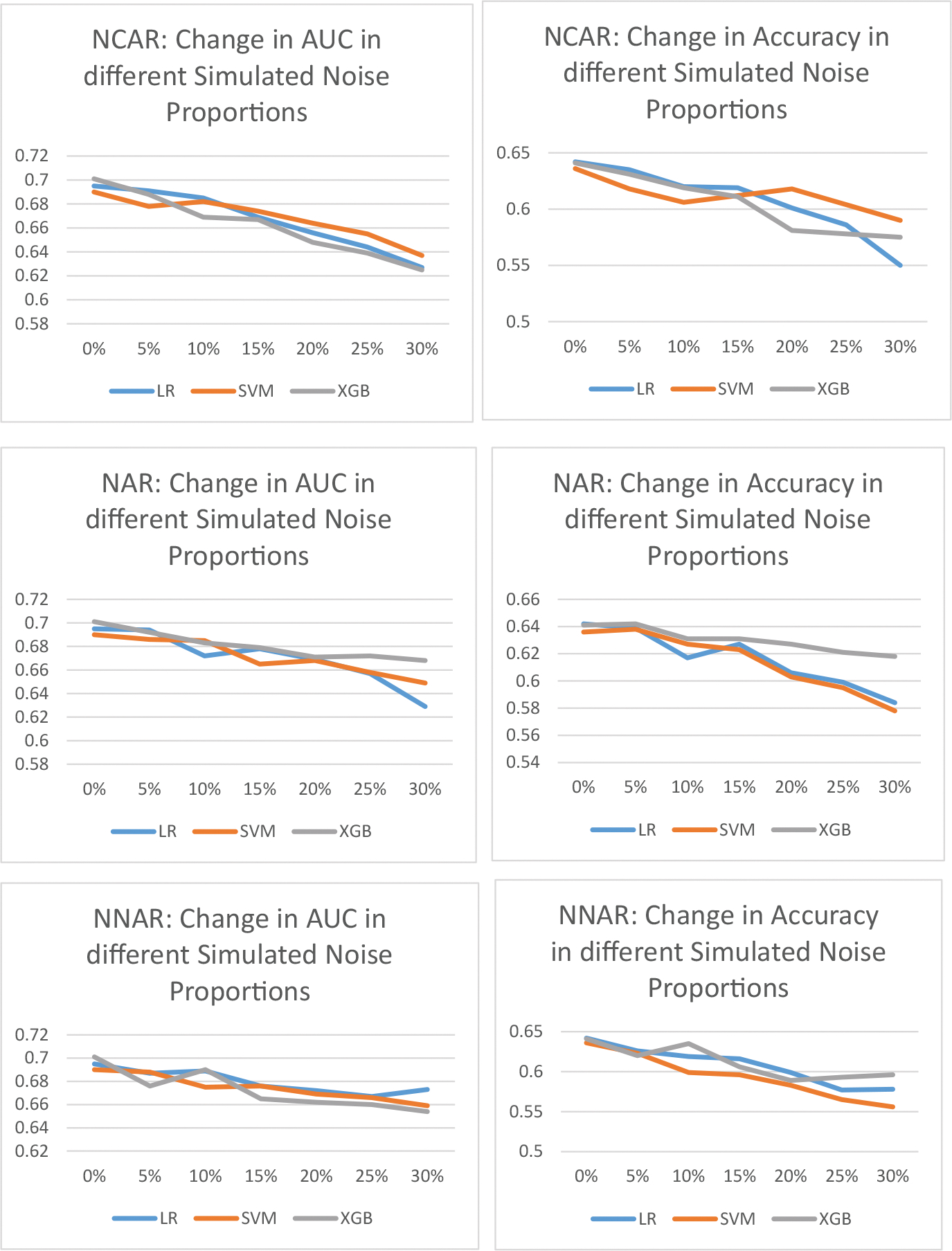
Change in model AUC and Accuracy as a function of noise proportion (0%, 5%, …, 30%) and noise generation mechanism (NCAR, NAR, NNAR).

Figure 1 shows the effect of different noise proportions crossed by noise generation mechanisms on the AUC and accuracy metrics. As Figure 1 shows, as the proportion of random noise increases, both the AUC and accuracy scores drop. The exact amount of decrease varies, but the pattern is consistent across different noise mechanisms and machine learning algorithms. Tables B-1 through B-3 in Appendix B show the numerical results of the AUC and accuracy under different random noise simulation mechanisms for different machine learning algorithms used in this study.

### Effect on Coefficients and Impact Scores

**Figure 2:**
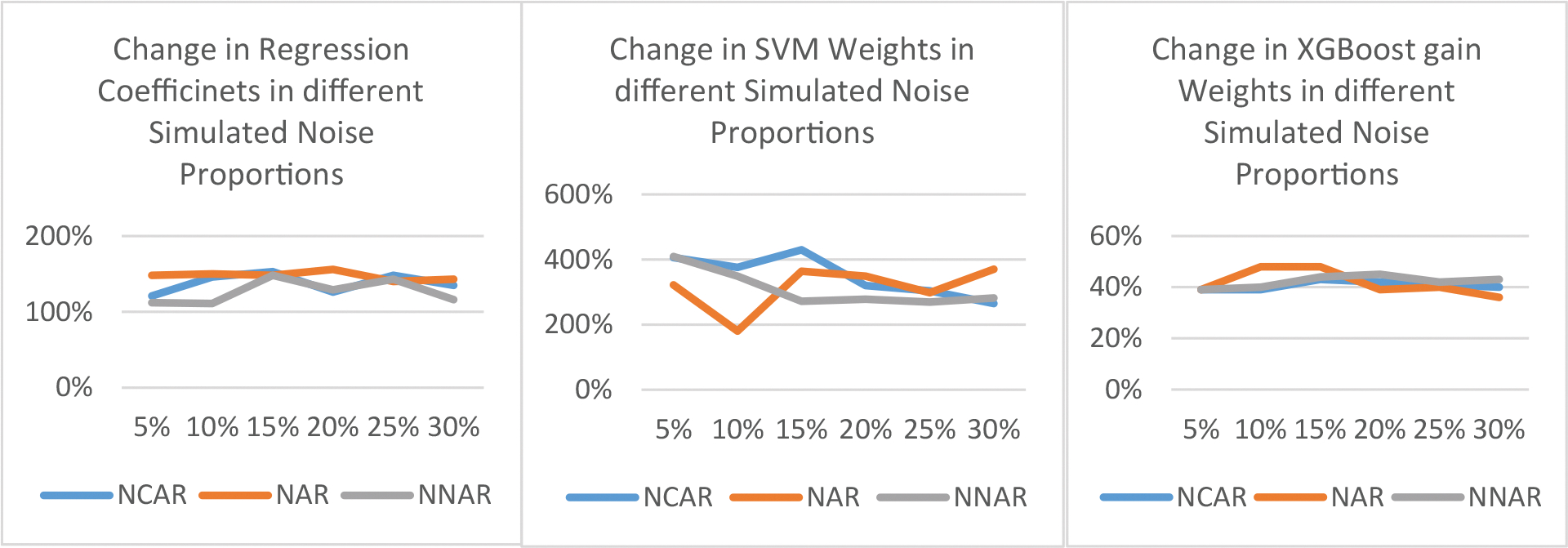
Percentage change in feature coefficients / weights as a function of different simulated noise generation mechanisms and proportions.

As shown in Figure 2, the coefficients in the logistic regression model change drastically, even at the 5% noise level. Similar drastic change patterns can be seen for the weights from SVM and XGBoost models (see Appendix C in the online supplement). The implication for research, and especially research replication is that even 5% of inconsistency or noise in data (through factors or covariates) can have a very noticeable effect. Equally alarming is the fact that the variance in the coefficients and impact scores diminish as more noise is added (see the distribution boxplots in Appendix D in the online supplement.)

In the examination of impact scores, the results of RM-ANOVA showed that the difference between various proportions of noise is not statistically significant (for all experiments, F < 2.6, p > 0.05). A closer examination of the 10 variables with the largest impact scores from logistic regression (which are the same values as the coefficients) reveals that the changes on the individual variable level are very concerning (Table 1 and Figure 3). At 5% noise level, all impact scores dropped sharply, and their relative ranks altered. We also observed such changes in SVM and in XGboost. (see Appendix E in the online supplement.)

## Discussion

In this study, our goal was to investigate the effect of noise in clinical data, such as data from EHR databases, on the prediction performance of different predictive machine learning models, including logistic regression, SVM, and gradient boosting. We mimicked variation of data quality at different levels by adding different proportions of random and non-random binary noise to existing data curated from the All of Us database. Since it is often impossible to determine the noise level in real world clinical data, we simulated noise in a postulated gold standard in order to measure our outcomes and used three different noise generation mechanism to mimic the variability of data in EHRs.

The results of these analyses tell a consistent story. In every case, increasing amounts of noise lead to lower accuracy and a diminished AUC. This result is not surprising. With more noise it is increasingly difficult to detect the signal. Whether the “noise” is simply from a random occurrence or represents another variable not included in the analysis, the resultant uncertainty has a deleterious effect on our ability to detect a signal. Of note is that the diminished signal can come from any variable, not just those of particular significance. This finding suggests that ignoring the noise in covariates is not a good strategy.

The most remarkable finding is that the variance of the impact scores (see box plots in Appendix D) diminishes with increasing noise, while the mean of the scores does not appear to change. Essentially, the impact of each variable is altered with diminished impact on the result. The diminution of the impact factors, which measure the contribution of a variable on the outcome, is remarkable. This result suggests that even small amounts of noise can diminish the ability to distinguish important risk factors in a predictive model. As a result, we believe that with “noisy” data such as that found in EHRs, such measures as hazard ratios, accuracy, and AUC calculations are potentially misleading. The consistency of the findings using different machine learning methods and different randomization procedures suggests that the loss of sensitivity is not a problem of brittleness [41], but more of a problem in dealing with the inherent variability in the data.

Our study was limited by the necessity of developing a reliable gold standard, leading to the requirement for simulating data and its variability. We chose ADRD as our outcome arbitrarily; we believe that the same would be true for other predictions for other outcomes as well. In addition to the effects of variability on observational studies, it is worthwhile to consider the possibility of similar effects on clinical trials. While most clinical trials using a randomized control design are tightly controlled in hopes of reducing the amount of variability involved, the hope is that any uncontrolled variable will be randomly assigned to either trial or control. Diagnosis is an uncertain process [42,43] and small upstream variations may have tremendous effects on the results of a study.

## Conclusion

While some data may be collected from well-controlled settings, there will inevitably be variations. As a result, the data used to make predictions about biological processes will always be variable. The findings of this study suggest that the predictions may be underestimating the effect of certain variables, and that ignoring uncertainties in the known covariates may lead to misleading conclusions. The little foxes of variability in data may indeed spoil the vineyard of reproducible results.

## Supplementary Material

Online Supplement

Please find the supplementary file here

https://www.fortunejournals.com/supply/fjhs13435.finalsupplement.pdf

## Figures and Tables

**Table 1:** Changes in 10 Largest Impact Scores of MCAR data with Logistic Regression

Block	noise00	noise05	noise10	noise15	noise20	noise25	noise30
117	0.98	0.366	0.101	0.02	0.103	0.072	0.07
63	0.876	0.434	0.33	0.305	0.125	0.099	0.021
34	0.856	0.367	0.195	0.141	0.021	0.013	0.035
2	0.826	0.047	0.119	0.094	0.057	0.022	0.051
106	0.745	0.638	0.501	0.461	0.293	0.3	0.187
175	0.728	0.052	0.055	0.041	0.028	0.022	0.083
9	0.691	0.16	0.113	0.021	0.039	0.094	0.094
71	0.608	0.046	0.121	0.079	0.005	0.035	0.021
67	0.599	0.234	0.01	0.113	0.027	0.045	0.052
171	0.579	0.062	0.034	0.099	0.002	0.124	0.113

## References

[1] Byrd JB, Vigen R, Plomondon ME, Data quality of an electronic health record tool to support VA cardiac catheterization laboratory quality improvement: the VA Clinical Assessment, Reporting, and Tracking System for Cath Labs (CART) program. Am Heart J. Mar 165 (2013): 434–40. 23453115 10.1016/j.ahj.2012.12.009

[2] Kahn MG, Callahan TJ, Barnard J, A Harmonized Data Quality Assessment Terminology and Framework for the Secondary Use of Electronic Health Record Data. EGEMS (Wash DC) 4 (2016): 1244. 27713905 10.13063/2327-9214.1244 PMC5051581

[3] Hohman KH, Klompas M, Zambarano B, Validation of Multi-State EHR-Based Network for Disease Surveillance (MENDS) Data and Implications for Improving Data Quality and Representativeness. Prev Chronic Dis. Jun 13 21 (2024): E43. 38870031 10.5888/pcd21.230409 PMC11192496

[4] Wassell M, Murray JL, Kumar C, Understanding Clinician EHR Data Quality for Reuse in Predictive Modelling. Stud Health Technol Inform. Jan 25 310 (2024): 169–173. 38269787 10.3233/SHTI230949

[5] Johnson SG, Speedie S, Simon G, A Data Quality Ontology for the Secondary Use of EHR Data. AMIA Annu Symp Proc. 2015 (2015): 1937–46. 26958293 PMC4765682

[6] Lee H, Lee Y, A Korean field trial of ICD-11 classification under practical clinical coding rules to clarify the reasons for inconsistencies. Health Inf Manag. Feb 24 (2025): 18333583251319371. 10.1177/18333583251319371 39995026

[7] Bailey MEA, Mistry J, Metcalfe A, An analysis of national variance in coding for patellofemoral instability. Knee. Dec 33 (2021): 386–92. 34781229 10.1016/j.knee.2021.10.023

[8] Endrich O, Rimle C, Zwahlen M, Asphyxia in the Newborn: Evaluating the Accuracy of ICD Coding, Clinical Diagnosis and Reimbursement. PLoS One. 12 (2017): e0170691. 28118380 10.1371/journal.pone.0170691 PMC5261744

[9] Hernando V, Sobrino-Vegas P, Burriel MC, Differences in the causes of death of HIV-positive patients in a cohort study by data sources and coding algorithms. AIDS. Sep 10 26 (2012): 1829–34. 22410685 10.1097/QAD.0b013e328352ada4

[10] Stevenson KB, Khan Y, Dickman J, Administrative coding data, compared with CDC/NHSN criteria, are poor indicators of health care-associated infections. Am J Infect Control. Apr 36 (2008): 155–64. 18371510 10.1016/j.ajic.2008.01.004

[11] Atolagbe OO, Romano PS, Southern DA, Coding rules for uncertain and “ruled out” diagnoses in ICD-10 and ICD-11. BMC Med Inform Decis Mak. Sep 27 21 (2024): 386. 39334213 10.1186/s12911-024-02661-6 PMC11430383

[12] Stewart CC, Simon G, Ahmedani BK, Variation in completeness of coding external cause of injuries under ICD-10-CM. Inj Prev. Jun 21 (2024). 10.1136/ip-2023-045164 PMC11662082 38906684

[13] Chung A, Opoku-Pare GA, Tibble H, Cause of death coding in asthma. BMC Med Res Methodol. Jun 5 24 (2024): 129. 38840045 10.1186/s12874-024-02238-x PMC11151540

[14] Ostrominski JW, Amione-Guerra J, Hernandez B, Coding Variation and Adherence to Methodological Standards in Cardiac Research Using the National Inpatient Sample. Front Cardiovasc Med. 8 (2021): 713695. 34796206 10.3389/fcvm.2021.713695 PMC8592936

[15] Horsky J, Drucker EA, Ramelson HZ, Accuracy and Completeness of Clinical Coding Using ICD-10 for Ambulatory Visits. AMIA Annu Symp Proc. 2017 (2017): 912–20. 29854158 PMC5977598

[16] O’Malley KJ, Cook KF, Price MD, Measuring diagnoses: ICD code accuracy. Health Serv Res. Oct 40 (2005): 1620–39. 16178999 10.1111/j.1475-6773.2005.00444.x PMC1361216

[17] Atolagbe OO, Romano PS, Southern DA, Coding rules for uncertain and “ruled out” diagnoses in ICD-10 and ICD-11. BMC Med Inform Decis Mak. Sep 27 21 (2024): 386. 39334213 10.1186/s12911-024-02661-6 PMC11430383

[18] Asensio Blasco E, Borrat Frigola X, Pastor Duran X, From Admission to Discharge: Leveraging NLP for Upstream Primary Coding with SNOMED CT. J Med Syst. May 22 49 (2025): 68. 40399485 10.1007/s10916-025-02200-4 PMC12095342

[19] Fatahi S, Vassileva J, Roy CK, Comparing emotions in ChatGPT answers and human answers to the coding questions on Stack Overflow. Front Artif Intell. 7 (2024): 1393903. 39351510 10.3389/frai.2024.1393903 PMC11439875

[20] Melot B, Drouet F, Alvarez C, Automated ICD10-Coding of Teleconsultations Conclusions in Primary Care. Stud Health Technol Inform. Oct 20 309 (2023): 135–36. 37869824 10.3233/SHTI230758

[21] Lee YM, Bacchi S, Sia D, Optimising vitrectomy operation note coding with machine learning. Clin Exp Ophthalmol. Aug 51 (2023): 577–84. 37221135 10.1111/ceo.14257

[22] Dong H, Falis M, Whiteley W, Automated clinical coding: what, why, and where we are? NPJ Digit Med. Oct 22 5 (2022): 159. 36273236 10.1038/s41746-022-00705-7 PMC9588058

[23] Karagounis S, Sarkar IN, Chen ES, Coding Free-Text Chief Complaints from a Health Information Exchange: A Preliminary Study. AMIA Annu Symp Proc. 2020 (2020): 638–47. 33936438 PMC8075463

[24] Dalloux C, Claveau V, Cuggia M, Supervised Learning for the ICD-10 Coding of French Clinical Narratives. Stud Health Technol Inform. Jun 16 270 (2020): 427–31. 32570420 10.3233/SHTI200196

[25] Bilal M, Hamza A, Malik N, NLP for Analyzing Electronic Health Records and Clinical Notes in Cancer Research: A Review. J Pain Symptom Manage. May 69 (2025): e374–e394. 39894080 10.1016/j.jpainsymman.2025.01.019

[26] Klug K, Beckh K, Antweiler D, From admission to discharge: a systematic review of clinical natural language processing along the patient journey. BMC Med Inform Decis Mak. Aug 29 24 (2024): 238. 39210370 10.1186/s12911-024-02641-w PMC11360876

[27] Chakravorti T, Koneru S, Rajtmajer S, Reproducibility and replicability in research: What 452 professors think in Universities across the USA and India. PLoS One. 20 (2025): e0319334. 40138274 10.1371/journal.pone.0319334 PMC11940819

[28] Predicting replicability of COVID-19 social science preprints. Nat Hum Behav. Feb 9 (2025): 248–49. 39706871 10.1038/s41562-024-01962-0

[29] Murphy J, Caldwell AR, Mesquida C, Estimating the Replicability of Sports and Exercise Science Research. Sports Med. Jun 16 (2025). 10.1007/s40279-025-02201-w PMC12513899 40522610

[30] Shannon CE, A mathematical theory of communication. Bell Syst Tech J. 27 (1948): 379–423.

[31] Nettleton DF, Orriols-Puig A, Fornells A, A study of the effect of different types of noise on the precision of supervised learning techniques. Artif Intell Rev. 33 (2010): 275–306.

[32] Xiao Z, Higgins S, The power of noise and the art of prediction. Int J Educ Res. 87 (2018): 36–46.

[33] Plevris V, Solorzano G, Bakas N, Investigation of performance metrics in regression analysis and machine learning-based prediction models. Presented at: 8th European Congress on Computational Methods in Applied Sciences and Engineering (ECCOMAS 2022); 2022; Oslo, Norway.

[34] Kolmar SS, Grulke CM, The effect of noise on the predictive limit of QSAR models. J Cheminform. 13 (2021): 92. 34823605 10.1186/s13321-021-00571-7 PMC8613965

[35] All of Us Research Program. https://allofus.nih.gov

[36] Nelson SJ, Yin Y, Trujillo Rivera EA, Are ICD codes reliable for observational studies? Assessing coding consistency for data quality. Digit Health. Jan-Dec 10 (2024): 20552076241297056. 39493629 10.1177/20552076241297056 PMC11528819

[37] Lamproudis A, Svenning TO, Torsvik T, Using a Large Open Clinical Corpus for Improved ICD-10 Diagnosis Coding. AMIA Annu Symp Proc. 2023 (2023): 465–73. 38222373 PMC10785868

[38] Cheng Y, Ahmed A, Zamrini E, Alzheimer’s Disease and Alzheimer’s Disease-Related Dementias in Older African American and White Veterans. J Alzheimers Dis. 75 (2020): 311–20. 32280090 10.3233/JAD-191188 PMC7306894

[39] Rubin DB, Inference and missing data. Biometrika. 63 (1976): 581–92.

[40] Shao Y, Cheng Y, Shah RU, Shedding Light on the Black Box: Explaining Deep Neural Network Prediction of Clinical Outcomes. J Med Syst. Jan 4 45 (2021): 5. 33404886 10.1007/s10916-020-01701-8 PMC7983057

[41] Geirhos R, Jacobsen J-H, Michaelis C, Shortcut learning in deep neural networks. Nat Mach Intell. 2 (2020): 665–73.

[42] Graber ML, Franklin N, Gordon R, Diagnostic error in internal medicine. Arch Intern Med. Jul 11 165 (2005): 1493–99. 16009864 10.1001/archinte.165.13.1493

[43] Kelly A, Panush RS, Diagnostic uncertainty and epistemologic humility. Clin Rheumatol. Jun 36 (2017): 1211–14. 28432522 10.1007/s10067-017-3631-8

