# Supplementary material for: Beware the Little Foxes that Spoil the Vines: Small Inconsistencies in Clinical Data Can Distort Machine Learning Findings
Source: Fortune J Health Sci. Author manuscript; Available in PMC 2026 Apr 17. (PMC13086067; doi:10.26502/fjhs.348)

Online supplement

Beware the Little Foxes That Spoil the Vines:

Small Inconsistencies in Clinical Data Can Distort Machine Learning Findings

This supplement to the paper includes additional tables and figures related to the simulation results that are not included in the main paper due to space limitation. This supplement includes the following five appendices:

Appendix A: ICD-9 and ICD-10 Code values and Code Blocks

Appendix B: Machine Learning Model Performance Metrics Exposed to Noisy Data

Appendix C: Percentage Change in Machine Learning Model Performance Exposed to Noisy Data

Appendix D: Distribution Plots of Impact Scores

Appendix E: Top 10 Code Blocks with Highest Change in Absolute Impact Score

Appendix A: ICD-9 and ICD-10 Code values and Code Blocks

Table A-1. ICD-9 and ICD-10 codes and their description to identify ADRD conditions

ICD Codes	Description	Diagnosis
290.0	SENILE DEMENTIA UNCOMP	Probable Dementia
290.10	PRESENILE DEMENTIA	Probable Dementia
290.40	VASCULAR DEMENTIA, UNCOMP	Probable Dementia
290.41	VASC DEMENTIA W DELIRIUM	Probable Dementia
290.42	VASC DEMENTIA W DELUSION	Probable Dementia
290.43	VASC DEMENTIA W DEPRESSN	Probable Dementia
291.2	Alcohol-induced persisting dementia	Probable Dementia
292.82	Drug-induced persisting dementia	Probable Dementia
294.10	Dementia w/o behave dist.	Probable Dementia

294.11	DEMENTIA W/BEHAV DISTURB	Probable Dementia
294.11/ 042	HIV DISEASE AND DEMENTIA W/BEHAV DISTURB	Probable Dementia
294.11/ 331.5	(IDIOPATHIC) NORMAL PRESSURE HYDROCEPHALUS AND DEMENTIA W/BEHAV DISTURB	Probable Dementia
294.11/ 332.0	PARKINSON'S DISEASE AND DEMENTIA W/BEHAV DISTURB	Probable Dementia
294.20	DEMENTIA UNSPEC, W/O BEHAV DISTURB	Probable Dementia
294.20/ 042	HIV DISEASE AND DEMENTIA UNSPEC, W/O BEHAV DISTURB	Probable Dementia
294.20/ 331.5	(IDIOPATHIC) NORMAL PRESSURE HYDROCEPHALUS AND DEMENTIA W/O BEHAV DISTURB	Probable Dementia
294.20/ 332.0	PARKINSON'S DISEASE AND DEMENTIA W/O BEHAV DISTURB	Probable Dementia
294.21	DEMENTIA UNSPEC, W BEHAV DISTURB	Probable Dementia
294.8	OTHER PERSISTENT MENTAL DISORDERS DUE TO CONDITIONS CLASSIFIED ELSEWHERE [“DEMENTIA NOS”]	Probable Dementia
331.0	ALZHEIMER'S DISEASE	Probable Dementia
331.19	FRONTOTEMP DEMENTIA NEC	Probable Dementia
331.2	SENILE DEGENERATION OF BRAIN	Probable Dementia
331.7	CEREBRAL DEGENERATION IN DISEASES CLASSIFIED ELSEWHERE	Probable Dementia
331.82	DEMENTIA W LEWY BODIES	Probable Dementia
331.89	OTHER CEREBRAL DEGENERATION	Probable Dementia
331.9	CEREBRAL DEGENERATION UNSPECIFIED	Probable Dementia
F01.50	VASCULAR DEMENTIA WITHOUT BEHAVIORAL DISTURBANCE	Probable Dementia
F01.51	VASCULAR DEMENTIA WITH BEHAVIORAL DISTURBANCE	Probable Dementia
F02.80	DEMENTIA IN OTHER DISEASES CLASSIFIED ELSEWHERE WITHOUT BEHAVIORAL DISTURBANCE	Probable Dementia
F02.80/ B20	HIV DISEASE AND DEMENTIA W/O BEHAV DISTURB	Probable Dementia
F02.80/ G10	HUNTINGTON'S DISEASE AND DEMENTIA W/O BEHAV DISTURB	Probable Dementia
F02.80/ G20	PARKINSON'S DISEASE AND DEMENTIA W/O BEHAV DISTURB	Probable Dementia

F02.80/ G91.2	(IDIOPATHIC) NORMAL PRESSURE HYDROCEPHALUS AND DEMENTIA W/O BEHAV DISTURB	Probable Dementia
F02.81	DEMENTIA IN OTHER DISEASES CLASSIFIED ELSEWHERE WITH BEHAVIORAL DISTURBANCE	Probable Dementia
F02.81/ B20	HIV DISEASE AND DEMENTIA W/BEHAV DISTURB	Probable Dementia
F02.81/ G10	HUNTINGTON'S DISEASE AND DEMENTIA W/BEHAV DISTURB	Probable Dementia
F02.81/ G20	PARKINSON'S DISEASE AND DEMENTIA W/BEHAV DISTURB	Probable Dementia
F02.81/ G91.2	(IDIOPATHIC) NORMAL PRESSURE HYDROCEPHALUS AND DEMENTIA W/BEHAV DISTURB	Probable Dementia
F03.90	UNSPECIFIED DEMENTIA WITHOUT BEHAVIORAL DISTURBANCE	Probable Dementia
F03.91	UNSPECIFIED DEMENTIA WITH BEHAVIORAL DISTURBANCE	Probable Dementia
F10.27	ALCOHOL DEPENDENCE WITH ALCOHOL-INDUCED PERSISTING DEMENTIA	Probable Dementia
F10.97	ALCOHOL USE, UNSPECIFIED WITH ALCOHOL-INDUCED PERSISTING DEMENTIA	Probable Dementia
F18.97	INHALANT USE, UNSPECIFIED WITH INHALANT-INDUCED PERSISTING DEMENTIA	Probable Dementia
F19.97	OTHER PSYCHOACTIVE SUBSTANCE USE, UNSPECIFIED WITH PSYCHOACTIVE SUBSTANCE-INDUCED PERSISTING DEMENTIA	Probable Dementia
G30.0	ALZHEIMER'S DISEASE WITH EARLY ONSET	Probable Dementia
G30.1	ALZHEIMER'S DISEASE WITH LATE ONSET	Probable Dementia
G30.8	OTHER ALZHEIMER'S DISEASE	Probable Dementia
G30.9	ALZHEIMER'S DISEASE, UNSPECIFIED	Probable Dementia
G31.09	OTHER FRONTOTEMPORAL DEMENTIA	Probable Dementia
G31.83	DEMENTIA WITH LEWY BODIES	Probable Dementia

Table A-2. ICD-10 codes grouped into Code Blocks

Code Block	From	To	Description
1	A00	A09	Intestinal infectious diseases
2	A15	A19	Tuberculosis
3	A20	A28	Certain zoonotic bacterial diseases
4	A30	A49	Other bacterial diseases
5	A50	A64	Infections with a predominantly sexual mode of transmission

6	A65	A69	Other spirochetal diseases
7	A70	A74	Other diseases caused by chlamydiae
8	A75	A79	Rickettsioses
9	A80	A89	Viral and prion infections of the central nervous system
10	A90	A99	Arthropod-borne viral fevers and viral hemorrhagic fevers
11	B00	B09	Viral infections characterized by skin and mucous membrane lesions
12	B10	B10	Other human herpesviruses
13	B15	B19	Viral hepatitis
14	B20	B20	Human immunodeficiency virus [HIV] disease
15	B25	B34	Other viral diseases
16	B35	B49	Mycoses
17	B50	B64	Protozoal diseases
18	B65	B83	Helminthiases
19	B85	B89	Pediculosis, acariasis and other infestations
20	B90	B94	Sequelae of infectious and parasitic diseases
21	B95	B97	Bacterial and viral infectious agents
22	B99	B99	Other infectious diseases
23	C00	C14	Malignant neoplasms of lip, oral cavity and pharynx
24	C15	C26	Malignant neoplasms of digestive organs
25	C30	C39	Malignant neoplasms of respiratory and intrathoracic organs
26	C40	C41	Malignant neoplasms of bone and articular cartilage
27	C43	C44	Melanoma and other malignant neoplasms of skin
28	C45	C49	Malignant neoplasms of mesothelial and soft tissue
29	C50	C50	Malignant neoplasms of breast
30	C51	C58	Malignant neoplasms of female genital organs
31	C60	C63	Malignant neoplasms of male genital organs
32	C64	C68	Malignant neoplasms of urinary tract
33	C69	C72	Malignant neoplasms of eye, brain and other parts of central nervous system
34	C73	C75	Malignant neoplasms of thyroid and other endocrine glands
35	C76	C80	Malignant neoplasms of ill-defined, other secondary and unspecified sites
36	C7A	C7A	Malignant neuroendocrine tumors
37	C7B	C7B	Secondary neuroendocrine tumors
38	C81	C96	Malignant neoplasms of lymphoid, hematopoietic and related tissue
39	D00	D09	In situ neoplasms
40	D10	D36	Benign neoplasms, except benign neuroendocrine tumors
41	D37	D48	Neoplasms of uncertain behavior, polycythemia vera and myelodysplastic syndromes
42	D3A	D3A	Benign neuroendocrine tumors
43	D49	D49	Neoplasms of unspecified behavior
44	D50	D53	Nutritional anemias
45	D55	D59	Hemolytic anemias
46	D60	D64	Aplastic and other anemias and other bone marrow failure syndromes
47	D65	D69	Coagulation defects, purpura and other hemorrhagic conditions

48	D70	D77	Other disorders of blood and blood-forming organs
49	D78	D78	Intraoperative and postprocedural complications of the spleen
50	D80	D89	Certain disorders involving the immune mechanism
51	E00	E07	Disorders of thyroid gland
52	E08	E13	Diabetes mellitus
53	E15	E16	Other disorders of glucose regulation and pancreatic internal secretion
54	E20	E35	Disorders of other endocrine glands
55	E36	E36	Intraoperative complications of endocrine system
56	E40	E46	Malnutrition
57	E50	E64	Other nutritional deficiencies
58	E65	E68	Overweight, obesity and other hyperalimentation
59	E70	E88	Metabolic disorders
60	E89	E89	Postprocedural endocrine and metabolic complications and disorders, not elsewhere classified
61	F01	F09	Mental disorders due to known physiological conditions
62	F10	F19	Mental and behavioral disorders due to psychoactive substance use
63	F20	F29	Schizophrenia, schizotypal, delusional, and other non-mood psychotic disorders
64	F30	F39	Mood [affective] disorders
65	F40	F48	Anxiety, dissociative, stress-related, somatoform and other nonpsychotic mental disorders
66	F50	F59	Behavioral syndromes associated with physiological disturbances and physical factors
67	F60	F69	Disorders of adult personality and behavior
68	F70	F79	Intellectual disabilities
69	F80	F89	Pervasive and specific developmental disorders
70	F90	F98	Behavioral and emotional disorders with onset usually occurring in childhood and adolescence
71	F99	F99	Unspecified mental disorder
72	G00	G09	Inflammatory diseases of the central nervous system
73	G10	G14	Systemic atrophies primarily affecting the central nervous system
74	G20	G26	Extrapyramidal and movement disorders
75	G30	G32	Other degenerative diseases of the nervous system
76	G35	G37	Demyelinating diseases of the central nervous system
77	G40	G47	Episodic and paroxysmal disorders
78	G50	G59	Nerve, nerve root and plexus disorders
79	G60	G65	Polyneuropathies and other disorders of the peripheral nervous system
80	G70	G73	Diseases of myoneural junction and muscle
81	G80	G83	Cerebral palsy and other paralytic syndromes
82	G89	G99	Other disorders of the nervous system
83	H00	H05	Disorders of eyelid, lacrimal system and orbit
84	H10	H11	Disorders of conjunctiva
85	H15	H22	Disorders of sclera, cornea, iris and ciliary body
86	H25	H28	Disorders of lens
87	H30	H36	Disorders of choroid and retina

88	H40	H42	Glaucoma
89	H43	H44	Disorders of vitreous body and globe
90	H46	H47	Disorders of optic nerve and visual pathways
91	H49	H52	Disorders of ocular muscles, binocular movement, accommodation and refraction
92	H53	H54	Visual disturbances and blindness
93	H55	H57	Other disorders of eye and adnexa
94	H59	H59	Intraoperative and postprocedural complications and disorders of eye and adnexa, not elsewhere classified
95	H60	H62	Diseases of external ear
96	H65	H75	Diseases of middle ear and mastoid
97	H80	H83	Diseases of inner ear
98	H90	H94	Other disorders of ear
99	H95	H95	Intraoperative and postprocedural complications and disorders of ear and mastoid process, not elsewhere classified
100	I00	I02	Acute rheumatic fever
101	I05	I09	Chronic rheumatic heart diseases
102	I10	I1A	Hypertensive diseases
103	I20	I25	Ischemic heart diseases
104	I26	I28	Pulmonary heart disease and diseases of pulmonary circulation
105	I30	I5A	Other forms of heart disease
106	I60	I69	Cerebrovascular diseases
107	I70	I79	Diseases of arteries, arterioles and capillaries
108	I80	I89	Diseases of veins, lymphatic vessels and lymph nodes, not elsewhere classified
109	I95	I99	Other and unspecified disorders of the circulatory system
110	J00	J06	Acute upper respiratory infections
111	J09	J18	Influenza and pneumonia
112	J20	J22	Other acute lower respiratory infections
113	J30	J39	Other diseases of upper respiratory tract
114	J40	J4A	Chronic lower respiratory diseases
115	J60	J70	Lung diseases due to external agents
116	J80	J84	Other respiratory diseases principally affecting the interstitium
117	J85	J86	Suppurative and necrotic conditions of the lower respiratory tract
118	J90	J94	Other diseases of the pleura
119	J95	J95	Intraoperative and postprocedural complications and disorders of respiratory system, not elsewhere classified
120	J96	J99	Other diseases of the respiratory system
121	K00	K14	Diseases of oral cavity and salivary glands
122	K20	K31	Diseases of esophagus, stomach and duodenum
123	K35	K38	Diseases of appendix
124	K40	K46	Hernia
125	K50	K52	Noninfective enteritis and colitis
126	K55	K64	Other diseases of intestines
127	K65	K68	Diseases of peritoneum and retroperitoneum

128	K70	K77	Diseases of liver
129	K80	K87	Disorders of gallbladder, biliary tract and pancreas
130	K90	K95	Other diseases of the digestive system
131	L00	L08	Infections of the skin and subcutaneous tissue
132	L10	L14	Bullous disorders
133	L20	L30	Dermatitis and eczema
134	L40	L45	Papulosquamous disorders
135	L49	L54	Urticaria and erythema
136	L55	L59	Radiation-related disorders of the skin and subcutaneous tissue
137	L60	L75	Disorders of skin appendages
138	L76	L76	Intraoperative and postprocedural complications of skin and subcutaneous tissue
139	L80	L99	Other disorders of the skin and subcutaneous tissue
140	M00	M02	Infectious arthropathies
141	M04	M04	Autoinflammatory syndromes
142	M05	M14	Inflammatory polyarthropathies
143	M15	M19	Osteoarthritis
144	M20	M25	Other joint disorders
145	M26	M27	Dentofacial anomalies [including malocclusion] and other disorders of jaw
146	M30	M36	Systemic connective tissue disorders
147	M40	M43	Deforming dorsopathies
148	M45	M49	Spondylopathies
149	M50	M54	Other dorsopathies
150	M60	M63	Disorders of muscles
151	M65	M67	Disorders of synovium and tendon
152	M70	M79	Other soft tissue disorders
153	M80	M85	Disorders of bone density and structure
154	M86	M90	Other osteopathies
155	M91	M94	Chondropathies
156	M95	M95	Other disorders of the musculoskeletal system and connective tissue
157	M96	M96	Intraoperative and postprocedural complications and disorders of musculoskeletal system, not elsewhere classified
158	M97	M97	Periprosthetic fracture around internal prosthetic joint
159	M99	M99	Biomechanical lesions, not elsewhere classified
160	N00	N08	Glomerular diseases
161	N10	N16	Renal tubulo-interstitial diseases
162	N17	N19	Acute kidney failure and chronic kidney disease
163	N20	N23	Urolithiasis
164	N25	N29	Other disorders of kidney and ureter
165	N30	N39	Other diseases of the urinary system
166	N40	N53	Diseases of male genital organs
167	N60	N65	Disorders of breast
168	N70	N77	Inflammatory diseases of female pelvic organs
169	N80	N98	Noninflammatory disorders of female genital tract

170	N99	N99	Intraoperative and postprocedural complications and disorders of genitourinary system, not elsewhere classified
171	Q00	Q07	Congenital malformations of the nervous system
172	Q10	Q18	Congenital malformations of eye, ear, face and neck
173	Q20	Q28	Congenital malformations of the circulatory system
174	Q30	Q34	Congenital malformations of the respiratory system
175	Q35	Q37	Cleft lip and cleft palate
176	Q38	Q45	Other congenital malformations of the digestive system
177	Q50	Q56	Congenital malformations of genital organs
178	Q60	Q64	Congenital malformations of the urinary system
179	Q65	Q79	Congenital malformations and deformations of the musculoskeletal system
180	Q80	Q89	Other congenital malformations
181	Q90	Q99	Chromosomal abnormalities, not elsewhere classified
182	S00	S09	Injuries to the head
183	S10	S19	Injuries to the neck
184	S20	S29	Injuries to the thorax
185	S30	S39	Injuries to the abdomen, lower back, lumbar spine, pelvis and external genitals
186	S40	S49	Injuries to the shoulder and upper arm
187	S50	S59	Injuries to the elbow and forearm
188	S60	S69	Injuries to the wrist, hand and fingers
189	S70	S79	Injuries to the hip and thigh
190	S80	S89	Injuries to the knee and lower leg
191	S90	S99	Injuries to the ankle and foot
192	T07	T07	Injuries involving multiple body regions
193	T14	T14	Injury of unspecified body region
194	T15	T19	Effects of foreign body entering through natural orifice
195	T20	T25	Burns and corrosions of external body surface, specified by site
196	T26	T28	Burns and corrosions confined to eye and internal organs
197	T30	T32	Burns and corrosions of multiple and unspecified body regions
198	T33	T34	Frostbite
199	T36	T50	Poisoning by, adverse effect of and underdosing of drugs, medicaments and biological substances
200	T51	T65	Toxic effects of substances chiefly nonmedicinal as to source
201	T66	T78	Other and unspecified effects of external causes
202	T79	T79	Certain early complications of trauma
203	T80	T88	Complications of surgical and medical care, not elsewhere classified

Appendix B: Machine Learning Model Performance Metrics Exposed to Noisy Data

Tables B-1 through B-3 show changes in model performance metrics (AUC and accuracy) of logistic regression (LR), support vector machines (SVM), and gradient boosting (XGB) machine learning algorithms under different proportions of simulated noise and noise generation mechanisms.

Table B-1: Change in AUC and accuracy as a function of the proportion of simulated noise following NCAR mechanism.

Noise	NCAR					
	AUC			Accuracy		
	LR	SVM	XGB	LR	SVM	XGB
00%	0.695	0.69	0.701	0.642	0.636	0.641
05%	0.691	0.678	0.688	0.635	0.618	0.631
10%	0.685	0.682	0.669	0.62	0.606	0.619
15%	0.669	0.674	0.667	0.619	0.612	0.611
20%	0.656	0.664	0.648	0.601	0.618	0.581
25%	0.644	0.655	0.639	0.586	0.604	0.578
30%	0.627	0.637	0.625	0.55	0.59	0.575

Table B-2: Change in AUC and accuracy as a function of the proportion of simulated noise following NAR mechanism.

Noise	NAR					
	AUC			Accuracy		
	LR	SVM	XGB	LR	SVM	XGB
00%	0.695	0.69	0.701	0.642	0.636	0.641
05%	0.694	0.686	0.692	0.639	0.638	0.642
10%	0.672	0.685	0.683	0.617	0.627	0.631
15%	0.678	0.665	0.679	0.627	0.623	0.631
20%	0.669	0.668	0.671	0.606	0.603	0.627
25%	0.657	0.658	0.672	0.599	0.595	0.621
30%	0.629	0.649	0.668	0.584	0.578	0.618

Table B-3: Change in AUC and accuracy as a function of the proportion of simulated noise following NNAR mechanism.

Noise	NNAR					
	AUC			Accuracy		
	LR	SVM	XGB	LR	SVM	XGB
00%	0.695	0.69	0.701	0.642	0.636	0.641
05%	0.687	0.688	0.676	0.626	0.623	0.62
10%	0.689	0.675	0.69	0.619	0.599	0.635
15%	0.676	0.676	0.665	0.616	0.596	0.606
20%	0.672	0.669	0.662	0.599	0.583	0.589
25%	0.667	0.666	0.66	0.577	0.565	0.593
30%	0.673	0.659	0.654	0.578	0.556	0.596

Appendix C: Percentage Change in Machine Learning Model Performance Exposed to Noisy Data

Tables C-1 through C-3 show the percent change in absolute feature weight across noise proportions and noise generation mechanisms for logistic regression (LR), support vector machines (SVM), and XGBoost (XGB) machine learning algorithms.

Table C-1: Percentage change in regression feature coefficients for different simulated noise proportions and mechanisms.

Noise	Percent Change in Regression Coefficients					
Mechanism	5%	10%	15%	20%	25%	30%
NCAR	121%	146%	153%	126%	148%	135%
NAR	148%	150%	148%	156%	140%	143%
NNAR	112%	111%	148%	129%	143%	116%

Table C-2: Percentage change in linear SVM feature weights for different simulated noise proportions and mechanisms.

Noise	Percent Change in linear kernel SCM Weights					
Mechanism	5%	10%	15%	20%	25%	30%
NCAR	406%	376%	430%	320%	304%	265%
NAR	322%	180%	364%	349%	298%	370%
NNAR	409%	349%	272%	278%	269%	282%

Table C-3: Percentage change in XGBoost feature gain weights for different simulated noise proportions and mechanisms.

Noise	Percent Change in linear kernel SCM Weights					
Mechanism	5%	10%	15%	20%	25%	30%
NCAR	39%	39%	43%	42%	41%	40%
NAR	39%	48%	48%	39%	40%	36%
NNAR	39%	40%	44%	45%	42%	43%

Appendix D: Distribution Plots of Impact Scores

Figures D-1 through D-9 show box plot distributions of the impact scores of variables modelled in logistic regression, linear SVM, and gradient boosting across different noise proportions and noise simulation mechanisms.

Figure D-1. Box plot of distribution of impact scores in logistic regression under random noise simulated by NCAR mechanism.

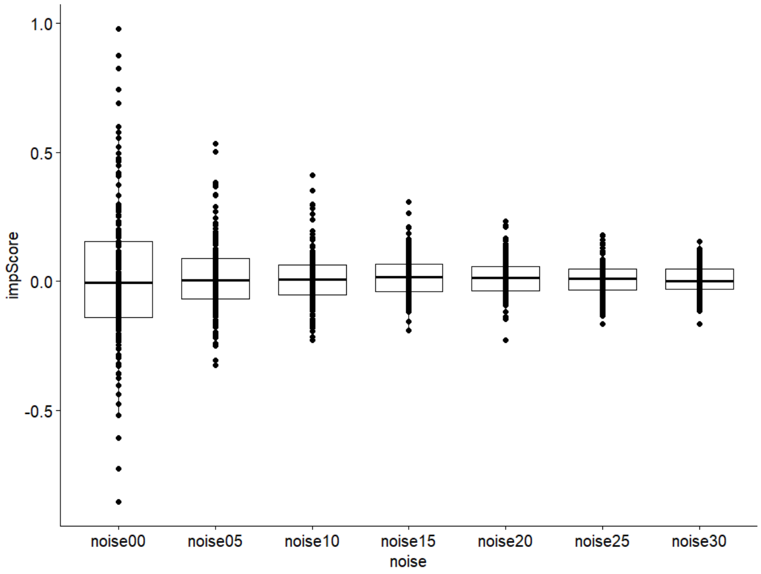

Figure D-2. Box plot of distribution of impact scores in logistic regression under random noise simulated by NAR mechanism.

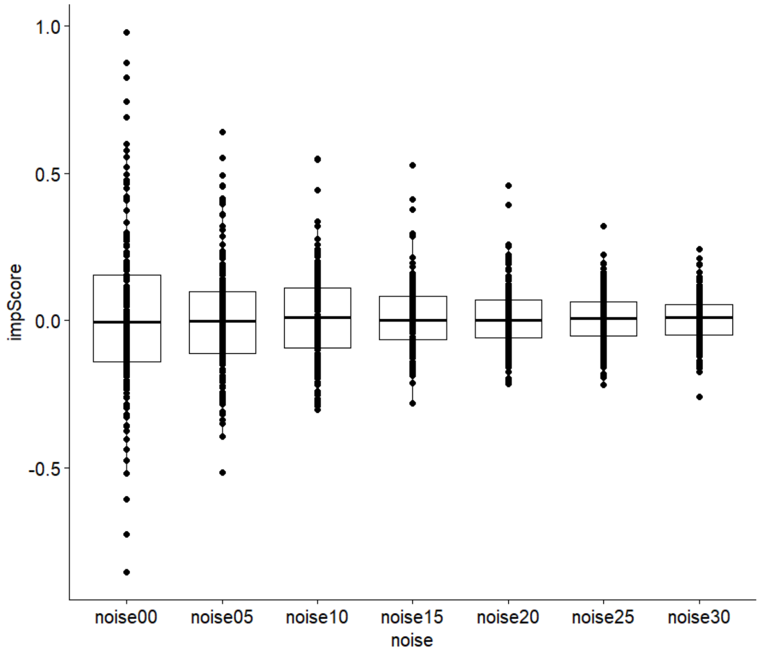

Figure D-3. Box plot of distribution of impact scores in logistic regression under random noise simulated by NNAR mechanism.

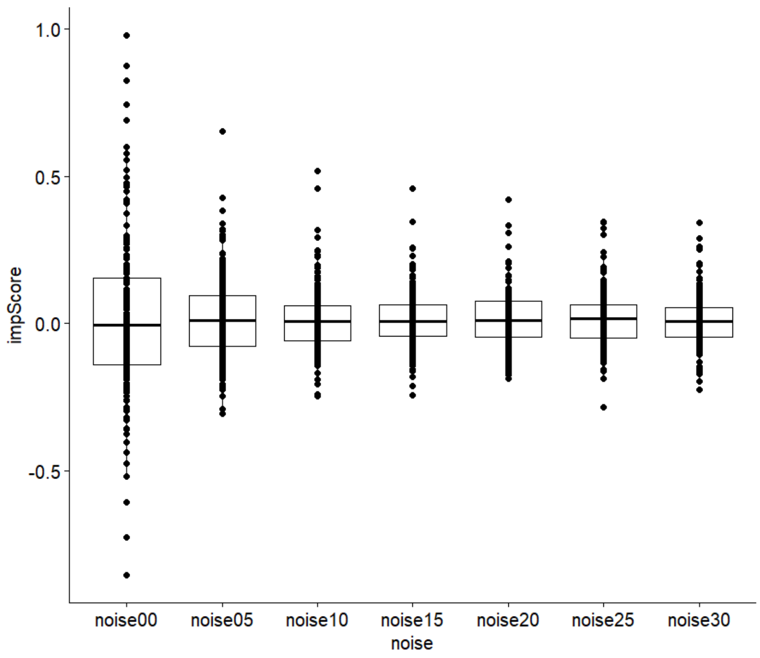

Figure D-4. Box plot of distribution of impact scores in SVM under random noise simulated by NCAR mechanism.

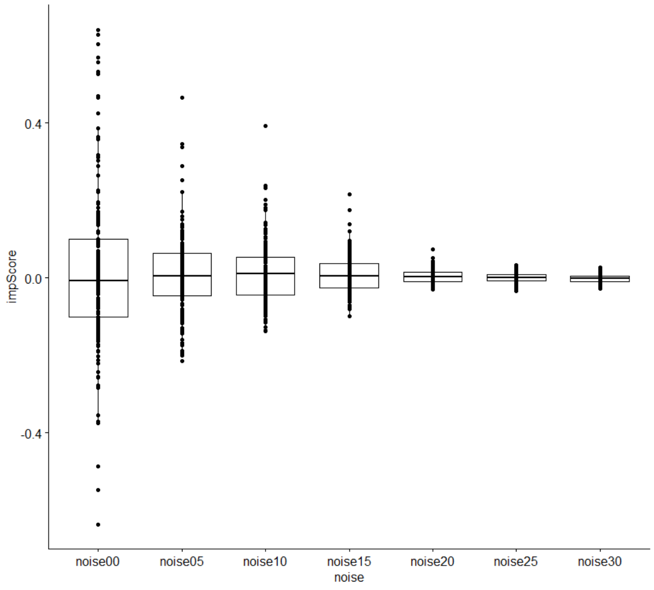

Figure D-5. Box plot of distribution of impact scores in SVM under random noise simulated by NAR mechanism.

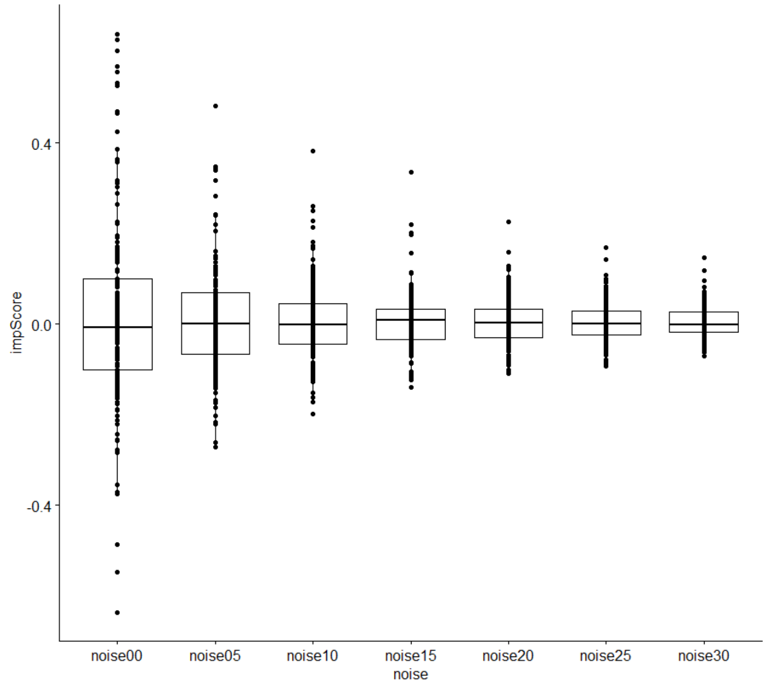

Figure D-6. Box plot of distribution of impact scores in SVM under random noise simulated by NNAR mechanism.

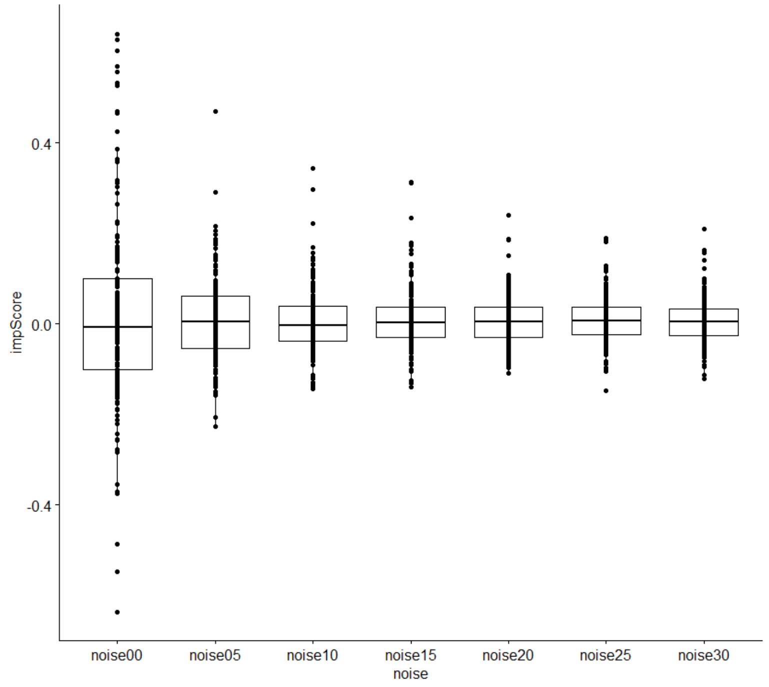

Figure D-7. Box plot of distribution of impact scores in gradient boosting under random noise simulated by NCAR mechanism.

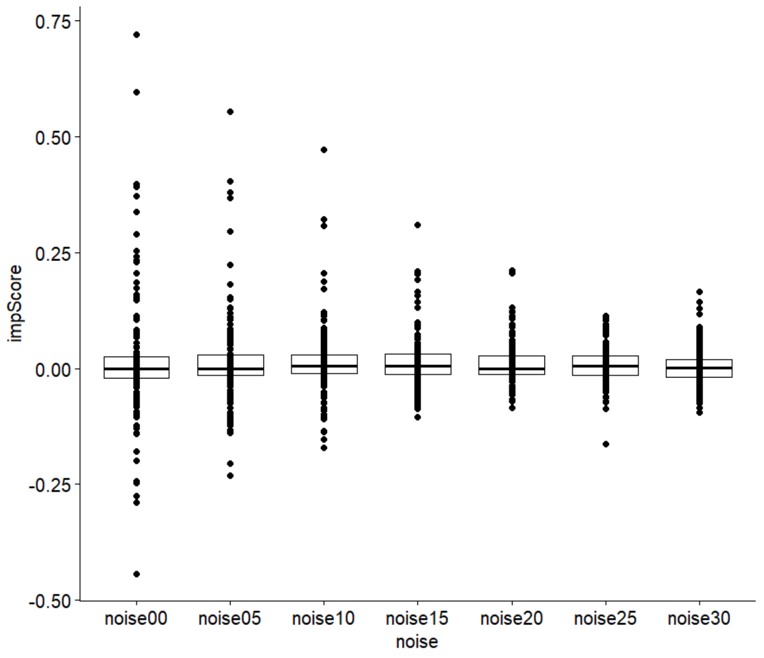

Figure D-8. Box plot of distribution of impact scores in gradient boosting under random noise simulated by NAR mechanism.

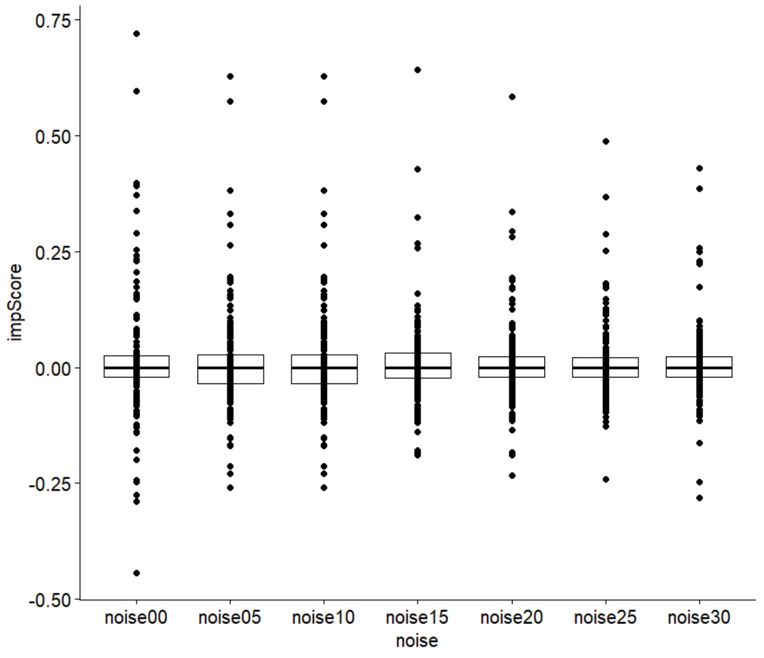

Figure D-9. Box plot of distribution of impact scores in gradient boosting under random noise simulated by NNAR mechanism.

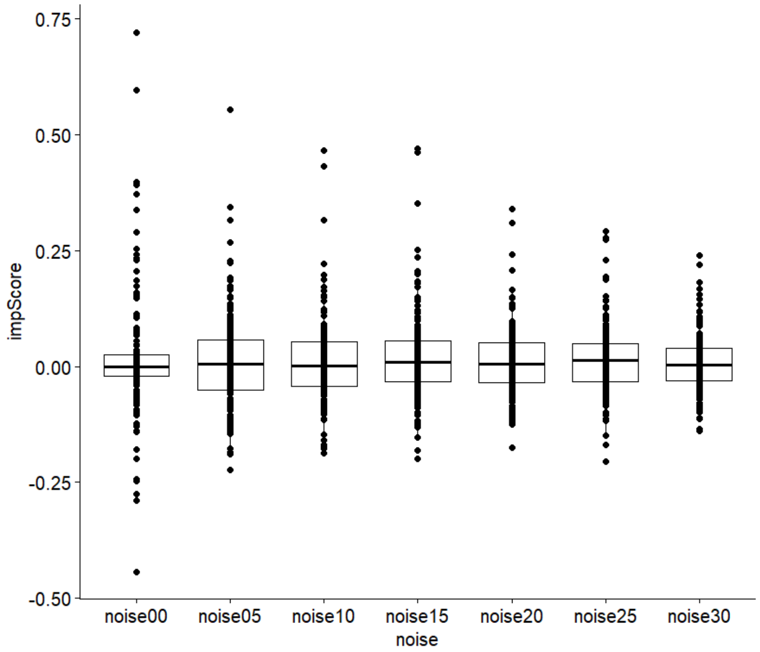

Appendix E: Top 10 Code Blocks with Highest Change in Absolute Impact Score

Tables E1-E6 and Figures E1-E6 show the top ten blocks with highest absolute change in impact scores across random noise proportion experiments and noise generation mechanisms.

Table E-1: Blocks with highest change in impact scores across noise proportions in NCAR mechanism.

Code Block	Noise 00	Noise 05	Noise 10	Noise 15	Noise 20	Noise 25	Noise 30
b117	0.980	0.366	0.101	0.020	0.103	0.072	0.070
b63	0.876	0.434	0.330	0.305	0.125	0.099	0.021
b34	0.856	0.367	0.195	0.141	0.021	0.013	0.035
b2	0.826	0.047	0.119	0.094	0.057	0.022	0.051
b106	0.745	0.638	0.501	0.461	0.293	0.300	0.187
b175	0.728	0.052	0.055	0.041	0.028	0.022	0.083
b9	0.691	0.160	0.113	0.021	0.039	0.094	0.094
b71	0.608	0.046	0.121	0.079	0.005	0.035	0.021
b67	0.599	0.234	0.010	0.113	0.027	0.045	0.052
b171	0.579	0.062	0.034	0.099	0.002	0.124	0.113

Figure E-1: Line plots of blocks with highest change in impact scores across noise proportions in NCAR mechanism

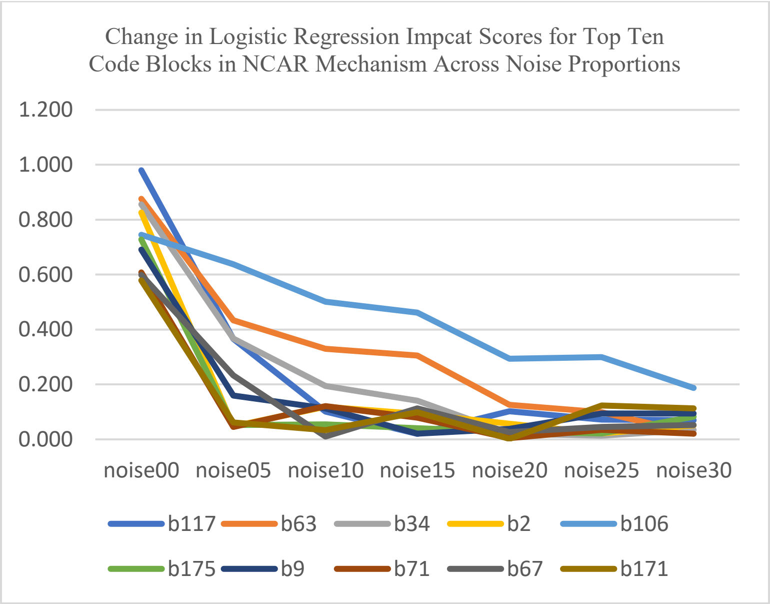

Table E-2: Blocks with highest change in impact scores across noise proportions in NAR mechanism.

Code block	Noise 00	Noise 05	Noise 10	Noise 15	Noise 20	Noise 25	Noise 30
b117	0.980	0.139	0.146	0.033	0.035	0.101	0.005
b63	0.876	0.521	0.317	0.230	0.281	0.079	0.110
b34	0.856	0.102	0.148	0.026	0.215	0.067	0.023
b2	0.826	0.194	0.005	0.151	0.048	0.026	0.020
b106	0.745	0.694	0.582	0.500	0.496	0.225	0.169
b175	0.728	0.128	0.076	0.074	0.119	0.008	0.005
b9	0.691	0.171	0.038	0.057	0.051	0.020	0.187
b71	0.608	0.209	0.002	0.080	0.089	0.023	0.051
b67	0.599	0.325	0.207	0.049	0.077	0.036	0.084
b171	0.579	0.200	0.106	0.082	0.062	0.004	0.067

Figure E-2: Line plots of blocks with highest change in impact scores across noise proportions in NAR mechanism

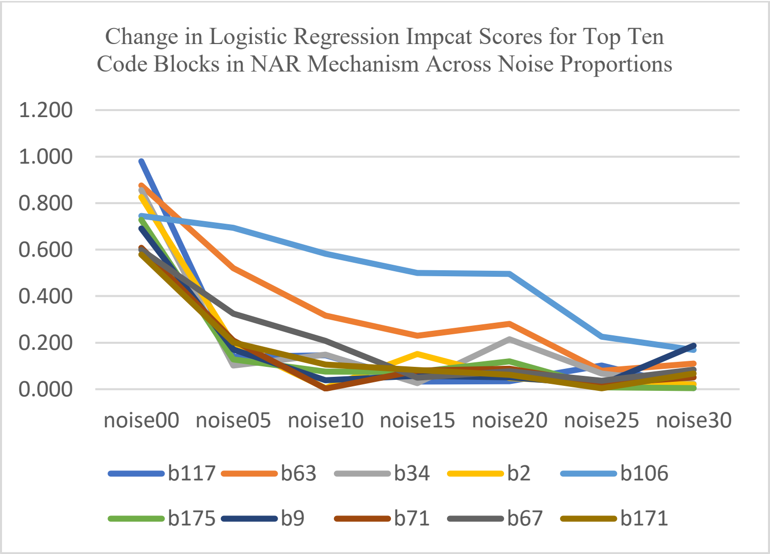

Table E-3: Blocks with highest change in impact scores across noise proportions in NNAR mechanism.

Code block	Noise 00	Noise 05	Noise 10	Noise 15	Noise 20	Noise 25	Noise 30
b117	0.980	0.070	0.083	0.035	0.013	0.002	0.062
b63	0.876	0.434	0.219	0.227	0.232	0.191	0.140
b34	0.856	0.110	0.075	0.097	0.164	0.118	0.047
b2	0.826	0.123	0.131	0.135	0.036	0.015	0.006
b106	0.745	0.620	0.505	0.463	0.438	0.371	0.436
b175	0.728	0.136	0.038	0.053	0.103	0.020	0.046
b9	0.691	0.253	0.007	0.119	0.021	0.044	0.059
b71	0.608	0.352	0.070	0.155	0.013	0.048	0.049
b67	0.599	0.238	0.136	0.091	0.145	0.070	0.107
b171	0.579	0.080	0.222	0.103	0.116	0.054	0.015

Figure E-3: Line plots of blocks with highest change in impact scores across noise proportions in NNAR mechanism

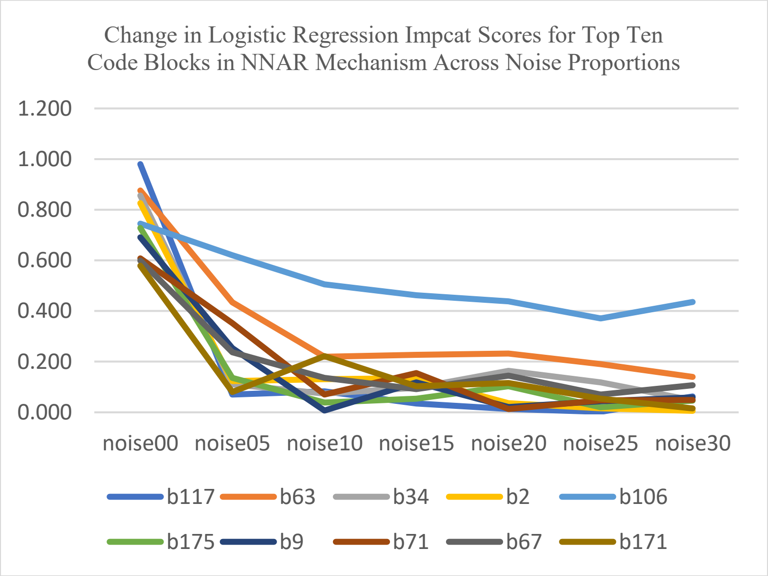

Table E-4: Blocks with highest change in impact scores across noise proportions in NCAR mechanism.

Code block	Noise 00	Noise 05	Noise 10	Noise 15	Noise 20	Noise 25	Noise 30
b117	0.640	0.101	0.001	0.018	0.012	0.000	0.000
b175	0.636	0.026	0.076	0.004	0.020	0.003	0.012
b53	0.628	0.037	0.049	0.027	0.027	0.015	0.017
b106	0.603	0.466	0.393	0.216	0.074	0.026	0.016
b17	0.569	0.056	0.086	0.027	0.008	0.020	0.009
b2	0.556	0.049	0.043	0.035	0.028	0.004	0.006
b34	0.548	0.092	0.003	0.007	0.019	0.001	0.004
b9	0.533	0.011	0.045	0.045	0.005	0.008	0.010
b63	0.528	0.338	0.174	0.050	0.023	0.003	0.015
b174	0.487	0.098	0.095	0.026	0.024	0.013	0.003

Figure E-4: Line plots of blocks with highest change in impact scores across noise proportions in NCAR mechanism

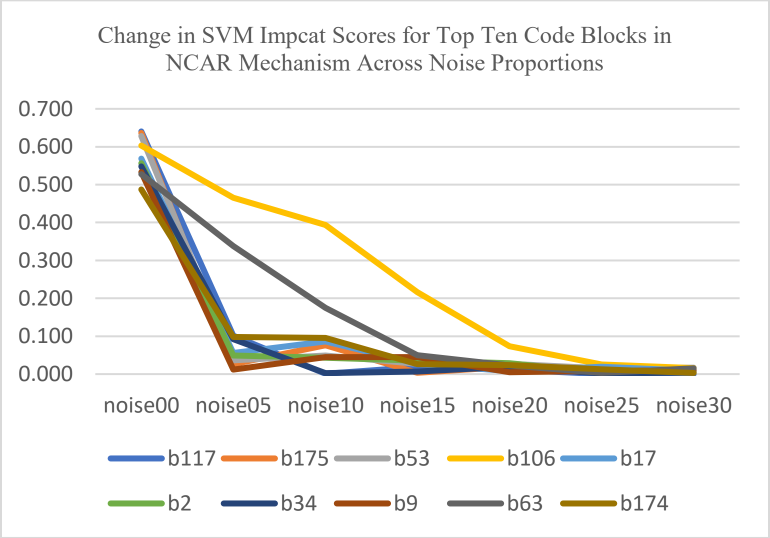

Table E-5: Blocks with highest change in impact scores across noise proportions in NAR mechanism

Code block	Noise 00	Noise 05	Noise 10	Noise 15	Noise 20	Noise 25	Noise 30
b117	0.640	0.027	0.015	0.112	0.002	0.077	0.059
b175	0.636	0.069	0.171	0.006	0.048	0.052	0.044
b53	0.628	0.048	0.101	0.025	0.005	0.025	0.044
b106	0.603	0.482	0.383	0.336	0.226	0.142	0.071
b17	0.569	0.205	0.023	0.072	0.096	0.071	0.029
b2	0.556	0.241	0.088	0.022	0.033	0.018	0.052
b34	0.548	0.079	0.121	0.049	0.043	0.029	0.038
b9	0.533	0.110	0.088	0.012	0.004	0.010	0.008
b63	0.528	0.348	0.250	0.197	0.125	0.099	0.082
b174	0.487	0.125	0.006	0.105	0.032	0.042	0.027

Figure E-5: Line plots of blocks with highest change in impact scores across noise proportions in NAR mechanism

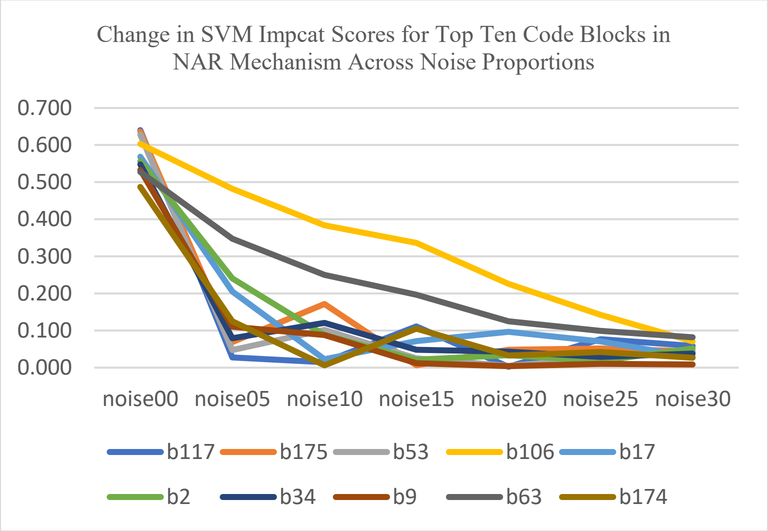

Table E-6: Blocks with highest change in impact scores across noise proportions in NNAR mechanism.

Code block	Noise 00	Noise 05	Noise 10	Noise 15	Noise 20	Noise 25	Noise 30
b117	0.640	0.002	0.039	0.019	0.009	0.001	0.002
b175	0.636	0.045	0.038	0.125	0.062	0.046	0.033
b53	0.628	0.030	0.010	0.090	0.012	0.030	0.009
b106	0.603	0.469	0.344	0.311	0.241	0.183	0.211
b17	0.569	0.084	0.009	0.075	0.001	0.077	0.062
b2	0.556	0.004	0.027	0.015	0.007	0.028	0.023
b34	0.548	0.072	0.130	0.002	0.078	0.023	0.068
b9	0.533	0.082	0.056	0.050	0.008	0.024	0.081
b63	0.528	0.176	0.141	0.116	0.047	0.091	0.033
b174	0.487	0.092	0.026	0.008	0.023	0.026	0.014

Figure E-6: Line plots of blocks with highest change in impact scores across noise proportions in NNAR mechanism

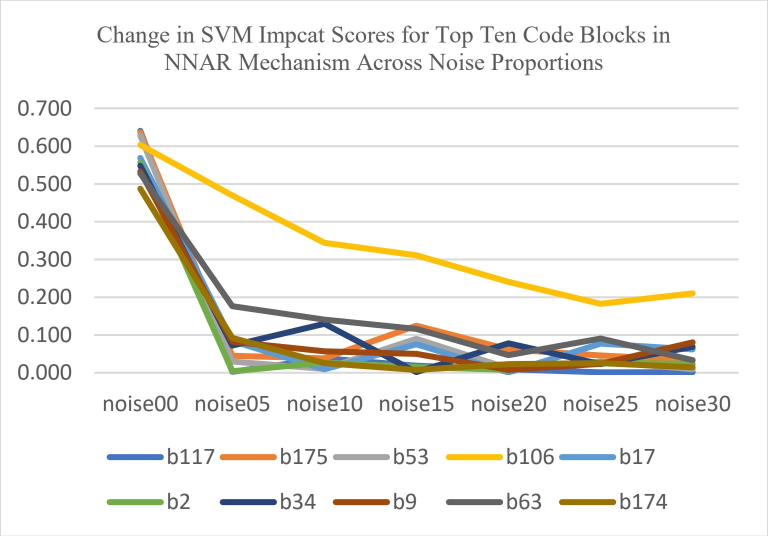

Table E-7: Blocks with highest change in impact scores across noise proportions in NCAR mechanism.

Code block	Noise 00	Noise 05	Noise 10	Noise 15	Noise 20	Noise 25	Noise 30
b63	0.721	0.368	0.172	0.144	0.028	0.013	0.016
b106	0.595	0.554	0.472	0.311	0.205	0.113	0.166
b34	0.442	0.019	0.001	0.005	0.008	0.014	0.008
b2	0.398	0.000	0.000	0.064	0.043	0.022	0.046
b67	0.391	0.296	0.118	0.055	0.014	0.002	0.048
b82	0.372	0.403	0.323	0.192	0.211	0.084	0.117
b64	0.338	0.379	0.307	0.210	0.111	0.050	0.008
b74	0.290	0.132	0.068	0.068	0.042	0.026	0.040
b167	0.289	0.139	0.063	0.021	0.039	0.025	0.052
b57	0.275	0.231	0.137	0.073	0.000	0.015	0.049

Figure E-7: Line plots of blocks with highest change in impact scores across noise proportions in NCAR mechanism

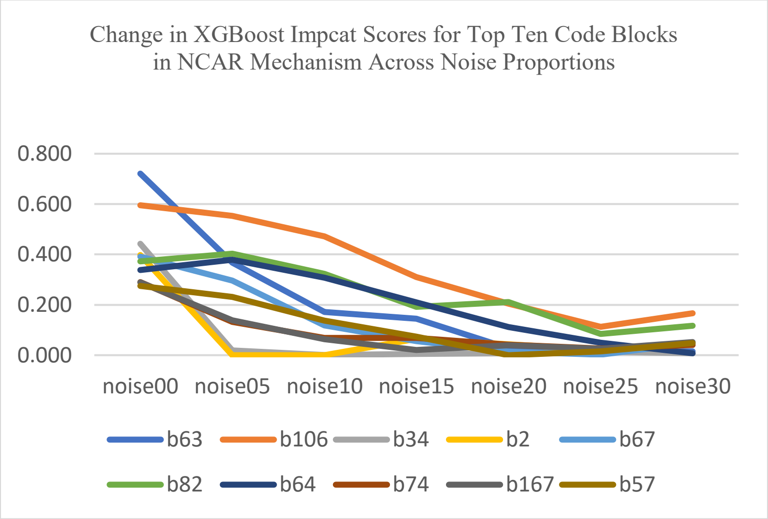

Table E-8: Blocks with highest change in impact scores across noise proportions in NAR mechanism.

Code block	Noise 00	Noise 05	Noise 10	Noise 15	Noise 20	Noise 25	Noise 30
b63	0.721	0.574	0.574	0.428	0.335	0.252	0.257
b106	0.595	0.627	0.627	0.642	0.585	0.488	0.430
b34	0.442	0.064	0.064	0.012	0.003	0.000	0.012
b2	0.398	0.192	0.192	0.037	0.016	0.016	0.032
b67	0.391	0.064	0.064	0.020	0.014	0.012	0.002
b82	0.372	0.383	0.383	0.268	0.282	0.182	0.173
b64	0.338	0.331	0.331	0.324	0.294	0.287	0.386
b74	0.290	0.307	0.307	0.258	0.187	0.368	0.230
b167	0.289	0.152	0.152	0.016	0.051	0.000	0.091
b57	0.275	0.260	0.260	0.189	0.183	0.240	0.024

Figure E-8: Line plots of blocks with highest change in impact scores across noise proportions in NAR mechanism

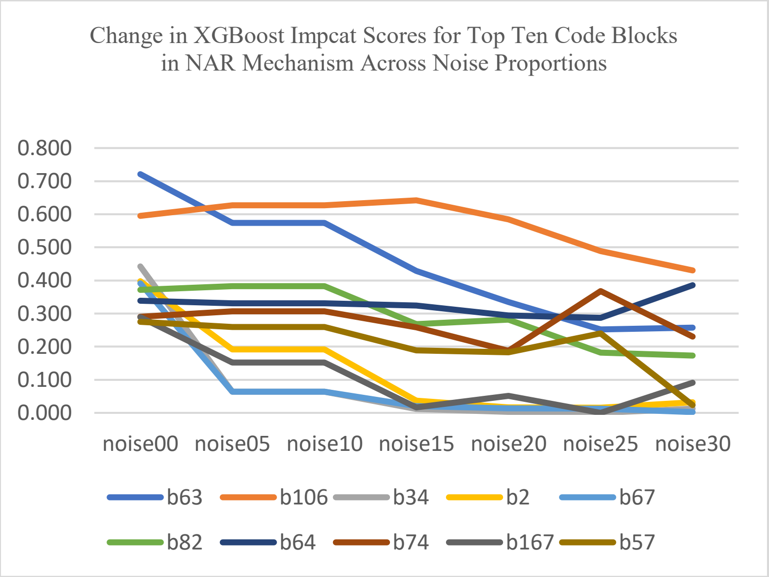

Table E-9: Blocks with highest change in impact scores across noise proportions in NNAR mechanism.

Code block	Noise 00	Noise 05	Noise 10	Noise 15	Noise 20	Noise 25	Noise 30
b63	0.721	0.165	0.164	0.200	0.033	0.111	0.036
b106	0.595	0.554	0.466	0.470	0.340	0.278	0.219
b34	0.442	0.064	0.159	0.014	0.097	0.041	0.056
b2	0.398	0.027	0.042	0.031	0.010	0.028	0.035
b67	0.391	0.173	0.187	0.206	0.049	0.045	0.060
b82	0.372	0.315	0.315	0.351	0.241	0.291	0.181
b64	0.338	0.343	0.431	0.463	0.309	0.274	0.239
b74	0.290	0.268	0.153	0.171	0.127	0.229	0.134
b167	0.289	0.143	0.169	0.105	0.061	0.013	0.043
b57	0.275	0.186	0.176	0.181	0.077	0.204	0.136

Figure E-9: Line plots of blocks with highest change in impact scores across noise proportions in NNAR mechanism

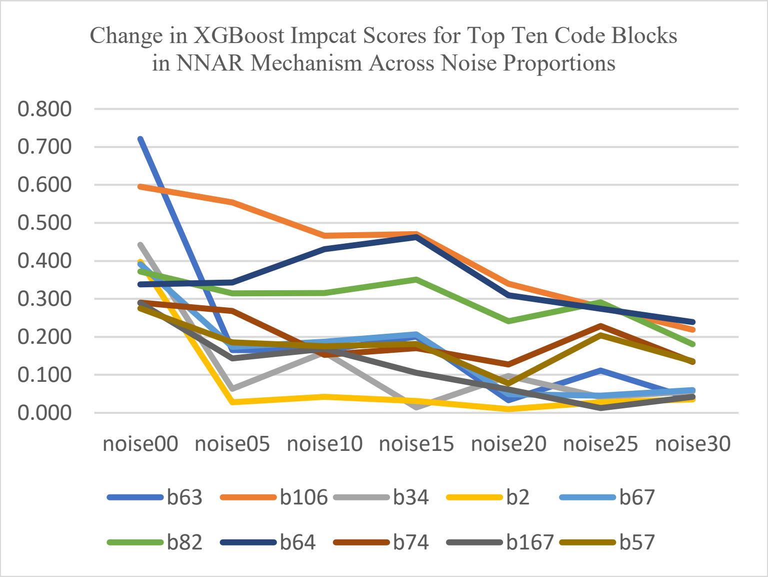

Supplement: Online Supplement [file NIHMS2152604-supplement-Online_Supplement.pdf]
